# 

**Published:** 1869-05

**Authors:** 


### DENTAL ASSOCIATION.

Mr. Editor: Permit me to call the attention of the delegates to, and members of, the American Dental Association, which holds its annual meeting at Saratoga, on the first Tuesday in August, to the desire intimated and expressed by some of making our next meeting additionally attractive and interesting, by combining the social elements with our professional gathering, and to this end the Committee of Arrangements would suggest and urge the delegates and members to bring their wives and daughters with them, in the hopes that by so doing additional interest will cluster around our gathering and add much to the pleasure and gratification to ourselves and those connected with us.

The committee will see that accommodations are provided for all who will give timely notice of their wishes by addressing the Chairman, stating what accommodations they require, &c.

J. G. Ambler, *Chairman*,<br>25 West Twenty-third St., New York.

### The Dental Register

### OF THE WEST.

*Issued Monthly, at $3 a Year in Advance.*

DEVOTED TO THE INTERESTS OF THE DENTAL PROFESSION.

EDITED BY J. TAFT AND G. WATT.

The volume commences in January and closes in December.

The Register being issued monthly is always fresh, therefore indispensable to the practitioner who desires to keep posted up with the new inventions and improvements which are daily developed. Neither expense nor effort will be spared to give value and variety to its contents. Articles will be illustrated with well executed engravings whenever they will add to the interest or assist in explanation.

Its extensive circulation and frequency of issue makes it one of the BEST DENTAL ADVERTISING MEDIUMS in the United States.

RATES OF ADVERTISING.

One page, 1 year, $50. Half page, 1 year, $30. Quarter page, or card, 1 year $20.

All advertising bills payable in advance, or at furthest after the issue of the first number containing the advertisement. All transient advertisements to be paid for in advance.

☞ CONTRIBUTIONS SOLICITED.

Address, J. TAFT, or "DENTAL REGISTER," Cincinnati Ohio.

Business Communications to

**J. TAFT, Publisher,**
No. 117 West Fourth St.

